# Measuring care trajectories using health administrative databases: a population-based investigation of transitions from emergency to acute care

**DOI:** 10.1186/s12913-016-1775-x

**Published:** 2016-10-11

**Authors:** John Paul Kuwornu, Lisa M. Lix, Jacqueline M. Quail, Xiaoyun Eric Wang, Meric Osman, Gary F. Teare

**Affiliations:** 1 Department of Community Health Sciences, College of Medicine, Faculty of Health Sciences, University of Manitoba, 750 Bannatyne Avenue, Winnipeg, MB R3E 0 W3 Canada; 2 Saskatchewan Health Quality Council, 111 Research Drive, Saskatoon, SK S7N 3R2 Canada

**Keywords:** Agreement, Data linkage, Emergency department, Healthcare trajectory, Hospital records

## Abstract

**Background:**

A patient’s trajectory through the healthcare system affects resource use and outcomes. Data fields in population-based administrative health databases are potentially valuable resources for constructing care trajectories for entire populations, provided they can capture patient transitions between healthcare services. This study describes patient transitions from the emergency department (ED) to other healthcare settings, and ascertains whether the discharge disposition field recorded in the ED data was a reliable source of patient transition information from the emergency to the acute care settings.

**Methods:**

Administrative health databases from the province of Saskatchewan, Canada (population 1.1 million) were used to identify patients with at least one ED visit to provincial teaching hospitals (*n* = 5) between April 1, 2006 and March 31, 2012. Discharge disposition from ED was described using frequencies and percentages; and it includes categories such as home, transfer to other facilities, and died. The kappa statistic with 95 % confidence intervals (95 % CIs) was used to measure agreement between the discharge disposition field in the ED data and hospital admission records.

**Results:**

We identified *N* = 1,062,861 visits for 371,480 patients to EDs over the six-year study period. Three-quarters of the discharges were to home, 16.1 % were to acute care in the same facility in which the ED was located, and 1.6 % resulted in a patient transfer to a different acute care facility. Agreement between the discharge disposition field in the ED data and hospital admission records was good when the emergency and acute care departments were in the same facility (κ = 0.77, 95 % CI 0.77, 0.77). For transfers to a different acute care facility, agreement was only fair (κ = 0.36, 95 % CI 0.35, 0.36).

**Conclusions:**

The majority of patients who attended EDs did not transition to another healthcare setting. For those who transitioned to acute care, accuracy of the discharge disposition field depended on whether the two services were provided in the same facility. Using the hospital data as reference, we conclude that the discharge disposition field in the ED data is not reliable for measuring transitions from ED to acute care.

## Background

A patient’s care trajectory [1], the patient’s sequence of contacts with care providers, is important to study because it is expected to affect resource use [2] and healthcare outcomes [3, 4]. However, there are no standard methods of constructing care trajectories. Population-based administrative health databases are potentially valuable resources for constructing care trajectories for entire populations, provided they can completely capture patient transitions between healthcare services. Incomplete capture of transitions between services will result in inaccurate calculations of healthcare outcomes such as costs, and this could have negative consequences for planning and allocation of healthcare resources [5].

Generally, a patient’s contacts with the healthcare system are kept in separate administrative health databases; including, for example, hospital records, physician billing claims, and emergency department (ED) data [6]. Some of these databases include data fields that define a patient’s previous and subsequent healthcare contacts. For example, the ED data contains fields that indicate the mode of arrival to and the discharge disposition from the department. These two data fields could be used to identify whether a patient utilized paramedic services prior to arriving in the ED and thereafter got admitted to inpatient acute care. Thus, it is conceivable that patient care trajectory spanning paramedic, ED, and inpatient acute care services can be constructed using data fields recorded in the ED database alone. Although this innovative use of data fields will eliminate the need for linking the ambulance, ED, and hospital data, the accuracy of data fields for capturing patient transitions between services are largely unknown.

Previous studies have focused on the overall quality of individual administrative health databases [6], and examined their utility for capturing patient data in single healthcare settings [7]. Only a few studies have compared multiple databases across settings, either by evaluating the accuracy of record linkage process for such data sources as ambulance, ED, and hospital [8], or by examining the completeness of integrated data sets to describe the patient’s journey through the healthcare system [9]. No study, to the best of our knowledge, has described the accuracy of data fields for capturing patient transition information between healthcare services.

This study investigated whether the discharge disposition field recorded in the ED data was a reliable source of patient transition information from the emergency to the acute care settings. Specifically, the objectives were to: (a) describe patient transitions from the ED to all other healthcare settings, and (b) estimate agreement between the discharge disposition field in the ED data and hospital admission records for capturing transitions from the emergency to the acute care settings. The ED often serves as the starting point for the receipt of services in the care trajectory, particularly among patients without a regular source of primary care [10]. ED encounters may require admission to hospital; one of the most expensive care settings. Thus, the ED and hospital are two of the most important healthcare services in describing patient care trajectories.

## Methods

### Data sources

ED data were obtained from all teaching hospitals (*n* = 5) in the province of Saskatchewan, Canada, which has a population of approximately 1.1 million [11]. This province, like other Canadian provinces, has a system of universal healthcare, which means that virtually all residents of the province are eligible for health insurance coverage. Only non-residents and individuals such as inmates in federal penitentiaries and members of the armed forces are not covered under the provincial insurance program. The teaching hospitals are located in Saskatoon and Regina Qu’Appelle health regions, two of 12 health regions in the province and the only regions that contain major urban centres (population > 200,000 in each centre). Three EDs started capturing patient data in electronic records in April 1, 2002, and by April 1, 2006 all five EDs were doing so. This study focused on the following fields of the electronic ED data: location, visit and discharge dates, and discharge disposition. Discharge disposition provides information on where the patient goes after treatment in the ED, and includes home, transfer to other facilities, left without being seen, and died. The relevant categories for describing transitions between ED and acute care were *admitted to the acute care hospital in which the ED was located* and *transferred for admission to an acute care hospital in a different facility*.

Electronic hospital discharge abstracts and population registry files were also used to conduct the research. A hospital discharge abstract is completed when a patient is discharged from an acute care facility. Hospital discharge data are available for all inpatient hospitalizations in the province. For this study, the relevant fields of the hospital discharge abstracts were the mode of entry to the hospital and the admission dates.

The population registry file contains demographic information, such as date of birth and residence location. It also captures dates of health insurance coverage.

All healthcare databases can be linked via a unique, anonymized personal health identification number. Data were accessed and analyzed at the provincial Health Quality Council in accordance with a standing data sharing agreement between the organization and the provincial ministry of health. Ethics approval for the research was received from the University of Saskatchewan Biomedical Research Ethics Board.

### Study cohort

The study adopted a population-based retrospective cohort design, which comprised all provincially insured individuals who had at least one visit to any of the five EDs between 2006/07 and 2011/12 fiscal years (a fiscal year extends from April 1 to March 31).

### Study measures

Patient characteristics selected for investigation were based on the Andersen healthcare utilization model [12], and included predisposing factors of age group (0–19, 20–39, 40–59, 60–79, 80+) and sex (male, female), and enabling factors of residence location (urban, rural) and health region affiliation (Saskatoon, Regina Qu’Appelle). Urban residents were those whose postal codes were in a census metropolitan or agglomeration area (i.e., 10,000+ population). All variables were measured as of the date of ED visit.

### Statistical analysis

To achieve the first study objective, the ED visit discharge dispositions were described using frequencies and percentages. To achieve the second objective, agreement between the discharge disposition field in the ED data and hospital admission records in capturing patient transitions between the two services was estimated using the kappa statistic (κ) [13]; 95 % confidence intervals (CIs) were also computed. The κ statistic has been used in previous studies to measure agreement between administrative health databases [14, 15]. We selected κ statistic as the measure of agreement because our variable of interest is binary (i.e., whether or not patient transitions from emergency care to acute care were recorded in both the ED and hospital databases) [16]. The value of κ is 1 when perfect agreement exists between two sources, 0 when agreement is equal to that expected assuming independence, and negative when agreement is less than expected by chance [17]. The interpretation of agreement adopted here is: poor (κ < 0.20), fair (κ = 0.20 to 0.39), moderate (κ = 0.40 to 0.59), good (κ = 0.60 to 0.79), and very good (κ = 0.80 to 1.00) [17]. When measuring agreement, we linked patient transitions from ED to acute care by matching hospital admissions which occurred on the same day as the ED discharge; as well as allowing up to 3 days between ED discharge date and hospital admission date in a sensitivity analysis [9]. Patient transitions between ED and acute care were assessed where the ED and the acute care hospital were in the same facility, as well as where the ED and the admitting acute care hospital were not in the same facility. Analyses were stratified by fiscal year and ED location.

Percentages (95 % CIs) were used to describe the differences between cohort members whose transition information was and was not missing. A patient’s transition information was considered missing if the ED discharge disposition indicated that the patient was admitted to an acute care hospital either in the same facility as the ED or in a different facility but the patient’s admission information was not recorded in the hospital discharge abstracts.

## Results

A total of 383,860 patients had at least one visit to an ED in the province’s teaching hospitals between 2006/07 and 2011/12 fiscal years. Of this number, 12,380 (3.2 %) patients did not have provincial insurance coverage (e.g., were not residents of the province) and were therefore excluded. Thus, the study cohort was comprised of 371,480 patients (96.8 %) with a total of 1,062,861 ED visits over the study period.

Table 1 summarizes the discharge dispositions for all ED visits by the study cohort members. Three-quarters of ED visits resulted in a discharge to the patient’s home, while 16.1 % resulted in admission to the acute care hospital in which the ED was located, and 1.6 % resulted in a transfer for admission to an acute care in a different facility.

**Table 1:** Emergency department discharge dispositions for the study cohort, April 1, 2006 to March 31, 2012

Discharge disposition	*n* (%)
Home	795,823 (74.9)
Admitted to the acute care hospital in which the ED was located	170,584 (16.1)
Unplanned discharge (i.e., left before being seen, left against medical advice after being seen by a doctor, signed out)	61,274 (5.7)
Transferred for admission to an acute care hospital in a different facility	16,951 (1.6)
Institutional place of residence (e.g., long-term care, jail)	10,129 (1.0)
Transfer within same facility (i.e., day surgery, out-patient care)	3,482 (0.3)
Transfer to external non-acute care facility	3,407 (0.3)
Died	1,211 (0.1)
Total	1,062,861 (100.0)

Notes: *ED* emergency department

The contingency table statistics used to calculate the overall agreement between the ED data and hospital records are summarized in Table 2. Of the 170,584 visits recorded in the ED data with a discharge disposition of *admitted to the acute care hospital in which the ED was located* (Table 1), only 143,633 of these visits were found in the hospital discharge abstracts with the same admission date as the ED discharge date (cell A in the upper half of Table 2). Similarly, of the 16,951 visits recorded in the ED data with a discharge disposition of *transferred for admission to an acute care hospital in a different facility* (Table 1), only 6,633 of these visits were found in the hospital discharge abstracts with the same admission date as the ED discharge date (cell A in the lower half of Table 2).

**Table 2:** Contingency table statistics used to calculate overall agreement between emergency department and hospital records

ED visits admitted to the acute care hospital in which the ED was located			
		Hospital records	
		Admitted to attached hospital on same day as discharge from ED	Not admitted to attached hospital on same day as discharge from ED
ED Records	Admitted to acute care in the attached hospital	A: True positives *n* = 143,633	B: False positives *n* = 26,951
	Not admitted to acute care in the attached hospital	C: False negatives *n* = 41,243	D: True negatives *n* = 851,034
ED visits transferred to an acute care hospital in a different facility			
		Hospital records	
		Admitted to a different hospital on same day as discharge from ED	Not admitted to a different hospital on same day as discharge from ED
ED Records	Transferred to an acute care in a different facility	A: True positives *n* = 6,633	B: False positives *n* = 10,318
	Not transferred to an acute care in a different facility	C: False negatives *n* = 12,495	D: True negatives *n* = 1,033,415

Notes: *ED* emergency department

Table 3 provides the results for the assessment of patient transitions from ED to acute care settings, stratified by fiscal year. For the case where patients were admitted to acute care in the same facility in which the ED was located, the overall agreement between the ED and hospital records was good (κ = 0.77, 95 % CI = 0.77, 0.77). The agreement between the two data sources was lowest (κ = 0.55, 95 % CI = 0.54, 0.55) in 2006/07 fiscal year and increased steadily to the highest value (κ = 0.94, 95 % CI = 0.94, 0.94) in 2011/12 fiscal year. For ED discharges to acute care in a different facility, the overall agreement was only fair (κ = 0.36, 95 % CI = 0.35, 0.36); agreement varied by fiscal year, reaching its peak (κ = 0.40, 95 % CI = 0.39, 0.42) in 2009/10.

**Table 3:** Agreement between emergency department (ED) and hospital records for capturing patient transition from ED to acute care, stratified by fiscal year

	Transition from ED to the acute care hospital in which the ED was located	Transition from ED to acute care in a different facility
Fiscal Year^a^	*κ* (95 % CI)	*κ* (95 % CI)
Overall	0.77 (0.77, 0.77)	0.36 (0.35, 0.36)
2006/07	0.55 (0.54, 0.55)	0.33 (0.31, 0.34)
2007/08	0.67 (0.67, 0.68)	0.34 (0.33, 0.36)
2008/09	0.73 (0.73, 0.74)	0.36 (0.34, 0.38)
2009/10	0.83 (0.82, 0.83)	0.40 (0.39, 0.42)
2010/11	0.86 (0.86, 0.86)	0.34 (0.33, 0.35)
2011/12	0.94 (0.94, 0.94)	0.36 (0.34, 0.38)

Notes: *CI* confidence interval, *ED* emergency department

^a^A fiscal year extends from April 1 to March 31

**Fig. 1:**
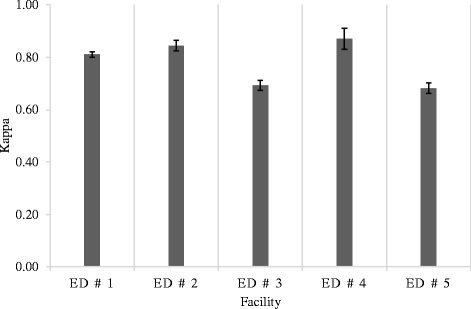
Agreement between emergency department (ED) and hospital records for capturing patient transition from ED to acute care, stratified by ED site. Note: The error bars represent 95 % confidence intervals

The level of agreement between ED and hospital records also varied across facilities (Fig. 1); with κ estimates ranging from good (i.e., κ = 0.68 for ED #5) to very good (i.e., κ = 0.87 for ED #4).

In the sensitivity analysis, when a lag of up to three days between ED discharge and hospital admission dates was allowed, agreement increased slightly both for admissions to acute care in the same facility in which the ED was located (from κ = 0.77 to κ = 0.80), and for transfers to an acute care hospital located in a different facility (from κ = 0.36 to κ = 0.43).

Table 4 compares the characteristics of cohort members whose transition information was and was not missing. Those whose information was missing from the ED to the acute care in which the ED was located had a total of 3,601 ED visits over the study period; and they were younger (62.8 % vs 38.6 % in the 20 to 59 years age group) and were more likely to be male (59.4 % vs 50.6 %) than those whose information was not missing. No major differences were observed between the two groups in terms of residence location and health region affiliation. Similarly, patients whose transition information was missing when they were transferred from the ED to an acute care in a different facility had a total of 1,218 ED visits over the study period; and were younger (64.1 % vs 43.3 % in the 20 to 59 years age group), and with more representation of males (61.9 % vs 49.7 %) than those whose information was not missing.

**Table 4:** Comparison of study cohort by status of transition information from emergency department to acute care

	Transition from ED to the acute care hospital in which the ED was located		Transition from ED to acute care in a different facility	
	Cohort without missing transition information from ED to the hospital in which the ED was located (*N* = 36,254)	Cohort with missing transition information from ED to the hospital in which the ED was located (*N* = 1,559)	Cohort without missing transition information from ED to a hospital located in a different facility (*N* = 3,742)	Cohort with missing transition information from ED to a hospital located in a different facility (*N* = 239)
% (95 % CI)				
Age group				
0–19	17.0 (16.7–17.3)	16.2 (14.5–18.1)	19.5 (18.3–20.8)	23.4 (18.5–29.2)
20–39	16.7 (16.5–16.9)	36.0 (33.6–38.4)	21.5 (20.3–22.9)	42.3 (34.6–46.9)
40–59	21.9 (21.6–22.1)	26.8 (24.6–29.0)	21.8 (20.5–23.2)	21.8 (17.0–27.4)
60–79	26.9 (26.6–27.1)	14.2 (12.5–16.0)	22.4 (21.0–23.7)	4.5 (2.6–8.1)
80+	17.5 (17.3–17.8)	6.8 (5.1–7.9)	14.8 (13.7–16.0)	8.0 (6.5–14.0)
Sex				
Female	49.4 (49.0–49.8)	40.6 (37.8–42.7)	50.3 (48.7–51.9)	38.1 (32.2–44.4)
Male	50.6 (50.3–50.9)	59.4 (56.9–61.8)	49.7 (48.1–51.3)	61.9 (54.4–66.7)
Residence location				
Urban	99.3 (99.3–99.4)	99.5 (98.9–99.7)	99.8 (99.6–99.9)	93.7 (89.9–96.2)
Rural	0.7 (0.7–0.8)	0.5 (0.3–1.0)	0.2 (0.1–0.4)	6.3 (3.8–10.1)
Health region				
Regina Qu’Appelle	50.5 (50.2–50.8)	50.1 (48.2–53.2)	48.0 (46.4–49.6)	54.4 (48.1–60.6)
Saskatoon	49.5 (49.1–49.9)	48.9 (46.5–51.4)	52.0 (50.4–53.6)	45.6 (39.4–51.9)

Notes: *ED* emergency department

## Discussion

The vast majority of patients who visited EDs in Saskatchewan’s teaching hospitals during the study period did not transition to other healthcare services; they were discharged home. Although the accuracy of the ED discharge to home information could not be ascertained within our study, our estimate of 74.9 % of ED visits being discharged home is consistent with another Canadian study which found 73.8 % of ED visits being discharged home during a similar time period [18]. Similarly, our results for discharges to acute care were quite similar to those recorded in the National Ambulatory Care Reporting System (NACRS) for Canada [19], especially for visits transferred for admission to an acute care in a different facility (average of 1.6 % over our study period versus an average of 1.1 % in NACRS over the same period). We found higher rates for admissions to the acute care hospital in which the ED was located than the NACRS data (16.1 % vs 9.4 %), and this might have occurred because ED visit rates reported in NACRS exclude scheduled visits.

The overall agreement between the discharge disposition field in the ED data and hospital admission records in capturing transitions from the ED to the acute care hospital in which the ED was located was good; a high proportion of all ED visits recorded as being discharged to an acute care hospital located within the same facility were identified in the hospital records as being admitted on the same date. We also noted a steady improvement in agreement between the two data sources over time. This may be an indication of improvements in data quality over time.

Among ED visits transferred to an acute care hospital located in a different facility, agreement between the discharge disposition field in the ED data and hospital admission records was only fair. Thus, a high proportion of all ED visits recorded as being discharged to an acute care hospital located in a different facility were not identified in the hospital records as being admitted to the facility on the same date. Also, the measure of agreement did not improve consistently over time for this category of ED discharges. The highest agreement was observed in 2009/10 fiscal year, and may be associated with a major outbreak of H1N1 flu virus in Canada [20]. A high level of inter-facility collaboration between the EDs and acute care hospitals likely occurred during this period.

Patient transition information between the ED and acute care were more likely to be missing at some ED locations than others. ED numbers 1 and 2 are located in the Regina Qu’Appelle health region whilst the other three EDs are located in Saskatoon health region. Although the data used in this study has been anonymized and harmonized by Saskatchewan eHealth, two separate information systems were used by the health regions for collecting the ED data. However, there was no clear influence of the difference in the information system on the results; since the EDs with the highest and lowest agreement were both located in the same health region. One of the EDs in Saskatoon health region was located in the biggest teaching hospital in the province and serves as the main trauma center for the province.

Allowing for up to three days between the ED discharge date and hospital admission date in a sensitivity analysis resulted in some improvement in agreement between the two data sources. The possibility of lags between ED discharge and hospital inpatient admission for the same episode of care was reported by a previous study [9]. Nonetheless, the overall agreement between the two data sources for ED visits discharged to an acute care hospital in a different facility remained low.

The low level of agreement between the discharge disposition field in the ED data and hospital admission records, particularly for ED visits discharged to acute care in a different facility, have implications for using data fields to construct care trajectories. Although the use of existing data fields is a simple approach than linking multiple databases to construct care trajectories, our findings indicate that these data fields could result in incomplete capture of patient transitions.

One potential reason for the low agreement between the discharge disposition field in the ED data and hospital admission records for ED discharges to acute care in a different facility is data entry errors. Peabody et al. [21] found inaccuracies in the coding of primary and secondary diagnosis in administrative data; similar coding errors might exist in the ED data with regards to the coding of the discharge disposition field. Another possible reason may be that patients did not reach the hospital to which they were being transferred, either due to death in the case of severely ill patients or a decision to seek care elsewhere. Further investigations, including chart reviews, could explore cause(s) of the low agreement between the two data sources in capturing patient transitions.

We were unable to evaluate the accuracy of discharge dispositions with regards to ED discharges to other types of care setting (i.e., non-acute care) because we did not have access to any linkable data sources containing these healthcare services. This study could be expanded to include healthcare utilizations prior to the arrival in ED such as ambulance services. By linking ambulance services, ED, and hospital data, future studies would be able to concurrently assess the accuracy of both the mode of entry and discharge disposition fields recorded in the ED data.

## Conclusion

In summary, the majority of patients who attended EDs during the study period did not transition to another healthcare setting. For those who transitioned to acute care, accuracy of the discharge disposition field depended on whether the two services were provided in the same facility. The discharge disposition field was more accurate in capturing transitions that occurred in the same facility than those that occurred between different facilities. Using the hospital data as reference, we conclude that the discharge disposition field recorded in the ED data did not capture the complete information on patient transitions to acute care services. Therefore, studies of patient care trajectories intended to describe transitions from the ED to acute care should not rely exclusively on the discharge disposition field, but rather be conducted by linking patient-specific records across the two care settings.

## Abbreviations

CI: Confidence intervals
ED: Emergency department
NACRS: National ambulatory care reporting system
